# Prevalence and associated factors of respiratory allergies in the Kingdom of Saudi Arabia: A cross-sectional investigation, September–December 2020

**DOI:** 10.1371/journal.pone.0253558

**Published:** 2021-06-23

**Authors:** Ahmad Almatroudi, Ayman M. Mousa, Divya Vinnakota, Adil Abalkhail, Ameen S. S. Alwashmi, Saleh A. Almatroodi, Fahad A. Alhumaydhi, Russell Kabir, Ilias Mahmud

**Affiliations:** 1 Department of Medical Laboratories, College of Applied Medical Sciences, Qassim University, Buraydah, Saudi Arabia; 2 Department of Basic Health Sciences, College of Applied Medical Sciences, Qassim University, Buraydah, Saudi Arabia; 3 Faculty of Medicine, Department of Histology and Cell Biology, Benha University, Benha, Egypt; 4 Faculty of Health, Education, Medicine and Social Care, School of Allied Health, Anglia Ruskin University, Chelmsford, Essex, United Kingdom; 5 Department of Public Health, College of Public Health and Health Informatics, Qassim University, Al Bukairiyah, Saudi Arabia; University of West London, UNITED KINGDOM

## Abstract

**Background:**

Prevalence of different respiratory allergies is increasing in the Kingdom of Saudi Arabia (KSA). Environmental risk factors of respiratory allergy vary regionally, hence the prevalence. This necessitates the needs for regional studies. This article reports prevalence and symptoms of respiratory allergies in the Qassim region, and the factors associated with the prevalence.

**Methods:**

Eight hundred and fifty individuals aged ≥18 years and were living in the Qassim region filled up our structured online questionnaire between September and December 2020. We estimated the prevalence of different respiratory allergies with 95% confidence intervals. Multi-variable logistic regression analyses were performed to investigate the risk factors of respiratory allergies.

**Findings:**

The prevalence of any respiratory allergy in the Qassim region was 28.8%. Most families (58.1%) had at least one member with respiratory allergy. The prevalence of allergic rhinitis and bronchial asthma were 13.5% and 11.2% 4.1% respectively. The reported symptoms included runny nose (13.6%), red, watery, and itchy eyes (10.4%), difficulty sleeping at night (10.2%), difficulty breathing in cold weather (9.2%), noisy breathing (8.5%), sneezing (8%), repeated coughing (7.5%) and shortness of breath (6.4%). Individuals with a family history were more likely to report any respiratory allergy (OR: 7.8), bronchial asthma (OR: 4.2) and allergic rhinitis (OR: 8.1) compared to the individuals without such family history. Odds of respiratory allergies was higher among males (OR: 1.5). Saudi nationals were less likely to report allergic rhinitis than the non-Saudis (OR: 0.4). Among those who reported a respiratory allergy, most (73.5%) received treatment and majority (61.7%) demonstrated compliance to the treatment, 8.8% needed hospitalization, and 23.1% needed emergency nebulization.

**Conclusions:**

Prevalence reported in our study is different than that reported in other regions. Variability in the environmental exposures might explain this. We recommend a meta-analysis to estimate the national prevalence of respiratory allergies.

## Introduction

Respiratory allergy is an extensive public health problem globally. The increasing incidence of allergies and allergic diseases are widely recognized worldwide [1]. About one-third of the world’s population has been affected by the allergic disorders [2]. Globally, 339 million people have asthma [3], 200 to 250 million have food allergy and 400 million have rhinitis [4]. There is a drastic increase in the prevalence of allergic respiratory diseases like rhinosinusitis and bronchial asthma because of environmental changes, industrialization, and immunologic interactions [5].

The prevalence of asthma is increasing in various high-income countries as well as in middle- and low-income countries [4]. Asthma is known as one among the most common chronic diseases in the Kingdom of Saudi Arabia (KSA), affecting more than 2 million people [6]. Globally, the most common non-communicable disease in children is asthma, and deaths occur mostly in older adults. Globally about 418 thousand people die of asthma annually and DALYS attributable to asthma are 24.8 million [3]. The main cause of respiratory allergies like allergic rhinitis and allergic asthma are the allergens from house dust mites [7]. House dust may contain dust mites, pollen, allergens from pets and particulates that can trigger respiratory allergies and asthma. Even climate changes can lead to increase in allergic diseases. Risk factors of asthma include a combination of genetic predisposition and exposure to the substances inhaled and the particulates that may irritate the airways and trigger allergic reactions. These particles can be smoke from tobacco, irritants from chemicals in work place, indoor and outdoor allergens, and air pollution [3]. Triggers for asthma can include temperature changes like cold air, emotions that are extreme like anger, fear, and physical exercise. Some specific medications like aspirin, NSAIDs and beta-blockers can also trigger asthma in certain conditions [3]. The impact of asthma can be in different ways. It does not only affect the patients but also families and the community. It is associated with missing school days, reduced quality of life, loss of work, frequent visits to emergency departments, increase in hospitalizations and deaths [8].

Recent studies have shown that prevalence of asthma is increasing rapidly than the prediction. The important factors that contribute to the burden of asthma in the KSA are poor knowledge, lack of awareness among primary care physicians caring for asthma patients in controlling the disease, and fear of using new drugs [9]. The KSA provides free health care services to its citizens but there is lack of research on the prevalence of respiratory allergies in the Qassim region, KSA. The Qassim region is an agricultural area in the centre of the KSA with a humid weather ranging from 50–70% which increases the incidence of allergic conditions and costs of medical care [10,11]. Mesquite is a small tree in the desert that has a potent pollen allergen, which is carried through sandstorms during spring and summer [12,13]. So, it is very important to get updated information on the prevalence, causes and management of respiratory allergies for prevention and proper management of respiratory allergic diseases in the KSA. This article reports the prevalence of different respiratory allergies and the predictors, perceived causes and treatment options explored by the patients for these allergies in the Qassim region, KSA.

## Material and methods

Ethical approval of this study was obtained from the ethics committee of the Deanship of Scientific Research, Qassim University. Approval number: CAMS1-2017-3-13-P-2225. First page of the online survey form contained informed consent statements. Participants could fill the online survey after they provided their informed consent.

### Study design

We employed a descriptive epidemiological method–cross-sectional survey to collect data on the prevalence of respiratory allergies, perceived causes, factors associated with the respiratory allergies and related health seeking behaviours. Study participants were recruited through online platforms (online groups and social media). Survey was conducted between September and December 2020.

### Study population and sampling

Any adult individual (aged ≥18 years), who was literate to respond to an online questionnaire in English or Arabic and was living in the Qassim region was invited to participate in this study through commonly used social media in the KSA. Since we disseminated our questionnaire through different social media, one might have received our invitation more than once. Therefore, we requested participants to avoid multi-registration. Sample size was calculated using the EpiInfo^TM^ 7. Keeping the population size as 999,999, assuming 50% prevalence of respiratory allergy, 95% confidence intervals and 80% power, the estimated minimum sample size was 384. That number was the highest possible number, even when the population size and the prevalence of respiratory allergies varied. However, by the end of our data collection period 850 participants filled our online survey form.

### The instrument and data collection

We used a structured questionnaire which was self-administered to the participants. The questionnaire was administered to a diverse community member in the Qassim region aged 18 year or over. The questionnaire was developed based on three questionnaires for screening of respiratory allergies, namely the Asthma Control Questionnaire to measure asthma control in adults, the International Study of Asthma and Allergies in Childhood and the European Community Respiratory Health Survey asthma questionnaire [14]. Our questionnaire contained six domains consisted of 19 major questions regarding the socio-demographic, diagnosis, risk factors, perceived causes, symptoms, and treatment measures of patients suffering from respiratory allergic diseases. The questionnaire was initially prepared in English, then translated into Arabic by two bilingual researchers, and compared with the original version by another researcher to get accurate English and Arabic versions.

We pre-tested the questionnaire before distribution to the participants. The pre-test involved 20 individuals and necessary modifications were made on wording and layout of the questionnaire based on pre-test experience. The questions were distributed through commonly used online social media platform such as twitter, WhatsApp, Instagram and snapchat. The questionnaire was also distributed through emails. The questionnaire took maximum five minutes to complete by the participants. First page of the online survey form contained the informed consent statements. Those provided consent had access to complete the questionnaire.

### Data analysis

We downloaded excel data file from the Google form. Data were then exported to the SPSS programme (Version 20 for Windows, SPSS Inc., Chicago, IL, USA) for analyses. Any participants who did not meet our inclusion criteria such as less than 18 years old, living outside the Qassim region were dropped from further analysis during the data cleaning process. We did descriptive analyses of socio-demographic variables, risk factors, perceived causes, self-reported respiratory allergy symptoms and health seeking behaviour variables. All variables were categorical or transformed into categorical variables (such as age group). We reported proportions for categorical variables. We estimated the prevalence of different respiratory allergies with 95% Confidence Intervals (CI). Multi-variable logistic regression analyses were performed to investigate the risk factors of respiratory allergies. We reported odds ratio (OR) with 95% CI for regression analyses. Hosmer and Lemeshow test (Chi-Square: value, *p value)*; Nagelkerke R Square value and corresponding *P* value were reported for regression analyses. *P* < 0.05 was considered statistically significant.

## Results

### Socio-demographics

Our study sample included 850 adults living in the Qassim region, KSA. Table 1 presents detailed socio-demographic information of the participants. We had a fair representation of both male and female genders among the participants. Most of the participants were aged 18–30 years (55.1%), living in the capital city of the Qassim region (67.1%), and Saudi nationals (95.2%), had a diploma or bachelor’s degree (65.1%), and were in paid employment (59.3%). Only 14.8% participants reported having present or history of smoking.

**Table 1:** Characteristics of the participants, prevalence, and predictors of respiratory allergies in Qassim, KSA, September–December 2020.

Characteristics	Percent (n)
**Gender**	
Male	52.2 (444)
Female	47.8 (406)
**Age**	
18–30 years	55.1 (468)
31–45 years	28.9 (246)
>45 years	16.0 (136)
**City**	
Buraidah	67.1 (570)
Unaizah	7.3 (62)
Alrass	6.7 (57)
Other	18.9 (161)
**Nationality**	
Saudi	95.2 (809)
Non-Saudi	4.8 (41)
**Educational attainment**	
Secondary or below	25.4 (216)
Diploma or bachelor	65.1 (553)
Postgraduate	9.5 (81)
**In paid employment**	
Yes	59.3 (504)
No	40.7 (346)
**Occupation related to the health sector**	
Yes	28.7 (244)
No	71.3 (606)
**Current or past history of smoking**	
Yes	14.8 (126)
No	85.2 (724)

### Prevalence of respiratory allergies

Tables 2 and 3 respectively presents self-reported prevalence of respiratory allergy symptoms and diagnosis of a respiratory allergy by health professionals. Reported symptoms included runny nose (13.6%), red, watery, and itching eyes (10.4%), difficulty breathing in cold weather (9.2%), noisy breathing (8.5%), sneezing (8.0%), repeated coughing (7.5%), difficulty sleeping at night (10.2%) and shortness of breath (6.4%).

**Table 2:** Self-reported prevalence of respiratory allergy symptoms in Qassim, KSA, September–December 2020.

Symptoms	Prevalence (95% CI)
Runny nose	13.6% (11.4%– 16.1%)
Red, watery, and itching eyes	10.4% (8.4%– 12.6%)
Difficulty breathing in cold weather	9.2% (7.3%– 11.3%)
Noisy breathing	8.5% (6.7%– 10.5%)
Sneezing	8.0% (6.3%– 10.0%)
Repeated coughing	7.5% (5.8%– 9.5%)
Difficulty sleeping	10.2% (8.3%– 12.5%)
Shortness of breath	6.4% (4.8%– 8.2%)

**Table 3:** Self-reported prevalence of diagnosed respiratory allergy in Qassim, KSA, September–December 2020.

Respiratory allergies	Prevalence (95% CI)
Any respiratory allergy	28.8% (25.8%– 32.0%)
Allergic rhinitis	13.5% (11.3%– 16.0%)
Bronchial asthma	11.2% (9.1%– 13.5%)
Other type of allergy	4.1% (2.9%– 5.7%)
Respiratory allergy in family	58.1% (54.7%– 61.5%)

The overall prevalence of any respiratory allergy in the Qassim region is 28.8%. Most families have at least one member with respiratory allergy (58.1%). The prevalence of allergic rhinitis, bronchial asthma and other types of respiratory allergies are 13.5%, 11.2% and 4.1% respectively.

### Predictors of respiratory allergies

Table 4 presents the predictors of respiratory allergies in the Qassim region, KSA. In multivariable logistic regression analysis, no evidence of significant association was observed between any respiratory allergies and socio-demographic variables, apart from gender with male are more likely to report any respiratory allergies (OR: 1.46, 95% CI:1.02–2.08), and present or past exposure to smoking. However, significant association was observed between any respiratory allergies and family history of respiratory allergies. The odds of any respiratory allergies among individuals with a family history of respiratory allergies are 7.8 (5.19–11.74) times the odds of any respiratory allergies among the individuals without such family history, adjusting for the effect of socio-demographic variables and smoking exposure.

**Table 4:** Predictors of respiratory allergies in Qassim, KSA, September–December 2020.

Characteristics	Any respiratory allergy	Bronchial asthma	Allergic rhinitis
	OR (95% CI)	OR (95% CI)	OR (95% CI)
**Gender**			
Male	**1.46 (1.02–2.08)**	1.59 (.99–2.56)	1.16 (.73–1.85)
Female	1	1	1
**Age**			
18–30 years	1.06 (.58–1.92)	1.48 (.62–3.54)	.73 (.35–1.52)
31–45 years	1.08(.65–1.81)	1.26 (.58–2.77)	.82 (.44–1.50)
>45 years	1	1	1
**City**			
Buraidah	1	1	1
Unaizah	.92 (.48–1.76)	1.09 (.43–2.76)	.74 (.33–1.66)
Alrass	1.62 (.83–3.16)	1.93 (.88–4.26)	.59 (.20–1.76)
Other	.74 (.48–1.14)	1.14 (.65–1.99)	.61 (.34–1.10)
**Nationality**			
Saudi	.75 (.35–1.62)	2.65 (.58–12.07)	**.40 (.17 –.93)**
Non-Saudi	1	1	1
**Educational attainment**			
Secondary or below	.98 (.66–1.45)	1.08 (.64–1.82)	.73 (.42–1.27)
Diploma, bachelor or above	1	1	1
**In paid employment**			
Yes	.86 (.53–1.41)	.82 (.43–1.59)	1.06 (.56–2.02)
No	1	1	1
**Current or history of smoking**			
Yes	1.48 (.90–2.43)	.87 (.43–1.75)	1.51 (.82–2.77)
No	1	1	1
**Family history of respiratory allergies**			
Yes	**7.80 (5.19–11.74)**	**4.15 (2.38–7.25)**	**8.11 (4.32–15.23)**
No	1		1
	Hosmer and Lemeshow test (Chi-square: 7.21, *p* = .514); Nagelkerke R Square: .210	Hosmer and Lemeshow test (Chi-square: 14.82, *p* = .063); Nagelkerke R Square: .092	Hosmer and Lemeshow test (Chi-square: 3.76, *p* = .878); Nagelkerke R Square: .169

We observed significant association between bronchial asthma and family history of bronchial asthma. No significant association between bronchial asthma and other socio-demographic variables and past or present history of smoking was evident. The odds of bronchial asthma among individuals with a family history of bronchial asthma is 4.15 (95% CI: 2.38–7.25) times the odds of bronchial asthma among the individuals without a family history, adjusting for the effects of socio-demographic variables and smoking exposure.

In multivariable logistic regression analysis, we observed evidence of significant association of allergic rhinitis with nationality and family history of allergic rhinitis. We found that the odds of allergic rhinitis among the individuals with a family history of allergic rhinitis are 8.11 (4.32–15.23) times the odds among the individuals without such family history, adjusting for the effects of socio-demographic variables and past or present history of smoking. We also found that Saudi nationals are less likely to report allergic rhinitis compared to non-Saudis (OR: .40, 95% CI: .17 –.93).

### Perceived causes of respiratory allergies

Among the participants who reported a diagnosed respiratory allergy, over a third (33.5%) did not know the cause, while the rest mentioned a range of causes including air pollution, smoking, atopy, respiratory tract infection, occupational exposure, diet, and genetic predisposition. Proportion of the participants reported these caused are presented in Table 5.

**Table 5:** Perceived causes of respiratory allergies in Qassim, KSA, September–December 2020.

Causes	Percent (n)
Air pollution	42.9 (105)
Smoking	11.0 (27)
Atopy	6.5 (16)
Respiratory tract infections	29.8 (73)
Occupational exposure	3.3 (8)
Diet	11.0 (27)
Genetic predisposition	20.8 (51)
Do not know	33.5 (82)

*multiple responses were allowed, n = 245.

### Health seeking behaviour for respiratory allergies

We found that among the respondents reported a confirmed diagnosis of respiratory allergy; most (73.5%) received treatment and majority (61.7%) demonstrated absolute compliance to the treatment provided by a health professional. Among the individual who sought treatment, 8.8% needed hospitalization, 23.1% needed emergency nebulization. Other provided treatment included inhaled bronchodilator spray, oral bronchodilator, inhaled steroid spray, oral steroid and IV steroid. Table 6 presents proportions of the participant given these treatments.

**Table 6:** Treatment of options explored by the people for respiratory allergies in Qassim, KSA, September–December 2020.

Treatment	Prevalence
Received/receiving treatment (n = 245)	73.5%
**Compliance to treatment**	
Always	61.7%
Sometime	29.0%
Never	9.3%
**Type of treatment**	
Inhaled bronchodilator spray	51.6%
Oral bronchodilator	36.3%
Inhaled steroid spray	28.6%
Oral steroid	10.4%
IV steroid	2.7%
Emergency nebulization	23.1%
Hospitalization	8.8%

*Multiple responses were allowed. n = 182 unless otherwise mentioned.

## Discussion

In this cross-sectional study, we estimated the prevalence of any respiratory allergies, bronchial asthma, allergic rhinitis, and other types of respiratory allergies. In addition, we have explored related health seeking behaviours. We have also estimated the prevalence of different self-reported symptoms of respiratory allergies. Furthermore, this article reports risk factors of these allergies in the Qassim region, KSA.

The findings from the study showed that the overall prevalence of any respiratory allergy in the Qassim region is 28.8%. The prevalence of allergic rhinitis and bronchial asthma are 13.5% and 11.2% respectively. We further found that in most families (58.1%) at least a member had respiratory allergy. Previous studies in the KSA, reported different prevalence estimates for respiratory allergies. According to a study conducted among medical students and interns, prevalence of asthma was 6.8% in the KSA [15]. An epidemiological study reported a noticeable increase in the prevalence of asthma from 8% in 1986 to 23% in 1995 among school children, aged 8–16 years in the KSA [16]. Another study, which investigated the prevalence of asthma among urban and rural children in the KSA, estimated the prevalence of asthma at 14.9% and 5.4% respectively [17]. Similarly, a study in the Abha region reported the prevalence as 9% [18], and a study in the Al Khobar city estimated the prevalence of asthma at 8.1% [19] among male school students. The rate of asthma reported in Saudi household survey 2013 was 4.05% [20]. In a nationwide study of asthma among adolescents in the KSA, the self-reported prevalence of asthma was found to be 8.2% [21]. The prevalence of allergic rhinitis is 13.5% in this study. This is lower that what reported by studies conducted in other parts of the KSA and in other countries. Allergic rhinitis affects 60 million people in the USA annually. Higher prevalence was observed among boys than girls and among women than men [22]. In a European study, prevalence of allergic rhinitis was about 25%, Italy (17%) and Belgium (28.5%) [23]. While a nationwide online survey in the KSA estimated the prevalence of intermittent and persistent allergic rhinitis at 54% and 46% respectively [24]. These differences in the estimated prevalence of respiratory allergies might be attributable to the variations in season, geography, and population groups covered by these studies. For example, a study in Finland reported that cold weather increases respiratory symptoms related to asthma and allergic rhinitis in young adults [25].

Runny nose was the most common symptom reported in our study compared to the other symptoms of respiratory allergies. Other symptoms reported by our study participants included red, watery, and itching eyes, difficulty breathing in cold weather, noisy breathing, sneezing, repeated coughing, difficulty sleeping and shortness of breath. This variability of symptoms respiratory allergy among patients is recognized in the literature and this variability makes classification based on the criteria proposed in guidelines on rhinitis and asthma challenging for the physicians [26]. The clinical manifestations of respiratory allergies are often related to the airborne allergens [26]. Therefore, a national updated guideline for classification and management of respiratory allergy diseases is necessary.

Our study found that males are almost two times likely to have bronchial asthma than the females. A study in Germany reported that prevalence of asthma between two genders varies by different age groups. Among the under 18 age group, the prevalence was higher in males than females. However, among the individuals aged 18 years or more the prevalence was higher among females than males [27]. Unlike Germany, in the KSA, due to cultural issues, males might have higher degree of exposure to different environmental risk factors of any respiratory allergies than the females. This perhaps explains the higher odds of any respiratory allergies among the males than the females in our study population.

Our study found that the family history of the disease plays an important role in asthma and allergic rhinitis development, individuals with family history of bronchial asthma and allergic rhinitis was over four times and eight times likely to have bronchial asthma and allergic rhinitis compared to the individuals without such family history, respectively. Similar findings are also reported in asthma literature. For example, as study reported that if a parent is having asthma, children is twice more likely to have asthma. The same study also reported that if a parent and grandparent have asthma, children are 4.3 times likely to have asthma. If only grandparent have asthma, grand children are 1.5 times likely to have asthma [28].

Unlike previous studies in different countries, which found evidence that individuals with lower educational attainment have a higher risk of developing asthma and other respiratory symptoms [29,30], and worse asthma morbidity in patients with asthma [31] we found lack of association between educational attainment and respiratory allergies. However, evidence suggests that individuals with lower educational attainment are associated with delayed health seeking for respiratory allergies [32], therefore might have resulted in underreporting in our study.

Among the study population who reported having a respiratory allergy, mentioned a range a cause. Causes reported by our study population included air pollution, smoking, atopy, respiratory tract infections, occupational exposure, diet, and genetic predisposition. However, one third of the participants did not know the cause. Having good knowledge about the cause of respiratory allergy can play a vital role in its prevention and management [33]. Our study population did not mention of the house dust mite which is a major perennial allergen source and a major cause of allergic rhinitis and allergic asthma [34]. However, awareness of the condition remains generally low in other population group too [34]. According to an Indian study the triggers of allergy following house dust are smoke and perfumes, 30.4% and 26.7% respectively [15].

The present study is first of its kind the Qassim region of the KSA and the findings will be influential in preparing longitudinal study with a larger sample size. There are some limitations of the study. First, the study was conducted only in the Qassim region, which results in cross-city limitations. In addition, online survey is unable to ensure random sampling. Therefore, our study population may not be representative. Nevertheless, higher than the required number of participants completed the survey and participants represented wider population groups. Second, the study was cross-sectional in nature and therefore we cannot conclude causality for the risk factors.

We found that more than a quarter of the adults in the Qassim region have respiratory allergies. This prevalence estimate is higher or lower than some previous studies in other regions in the KSA. Gender and family history of respiratory allergy were found to have an association with the prevalence of respiratory allergies. We recommend a meta-analysis of all prevalence studies conducted in the KSA to estimate the national prevalence of different respiratory allergies. While a long-term birth cohort studies could be useful to investigate the genetic, environmental, and behavioural risk factors of respiratory allergies in the KSA.

## Supporting information

S1 Appendix The Arabic questionnaire. (PDF)

S2 Appendix The English questionnaire. (PDF)

S3 Appendix The data file. (SAV)
