# Supplementary material for: Prevalence and associated factors of respiratory allergies in the Kingdom of Saudi Arabia: A cross-sectional investigation, September–December 2020
Source: PLoS One. 2021 Jun 23;16(6):e0253558. doi: 10.1371/journal.pone.0253558 (PMC8221486; doi:10.1371/journal.pone.0253558)
Supplement: S1 Appendix — (PDF) [file pone.0253558.s001.pdf]

استبيان الحساسية التنفسية بمنطقة القصيم في المملكة العربية السعودية

أسئلة حول المعلومات الشخصية

1. مكان السكن؟ * (اختر خيار واحد فقط)
    1. بريدة
    2. عنيزة
    3. الرس
    4. محافظة أخرى في منطقة القصيم
    5. خارج منطقة القصيم
2. الجنسية؟ *(اختر خيار واحد فقط)
    1. سعودي
    2. غير سعودي
3. الجنس؟ * (اختر خيار واحد فقط)
    1. ذكر
    2. أنثى
4. العمر (سنوات)؟ * (اختر خيار واحد فقط)
    1. أصغر من 15
    2. 15 - 30
    3. 31 - 45
    4. أكبر من 45
5. طبيعة العمل؟ * (اختر خيار واحد فقط)
    1. طالب
    2. موظف
    3. عامل
    4. وظيف أخرى

6. هل يرتبط عملك بقطاع صحي؟ * (اختر خيار واحد فقط)

   1. نعم
   2. لا

7. مستوى التعليم؟ * (اختر خيار واحد فقط)

   1. تعليم ماقبل الجامعي
   2. دبلوم أو بكالوريوس
   3. دراسات عليا

8. هل أنت مدخن حالياً أو كنت مدخن في السابق؟ * (اختر خيار واحد فقط)

   1. نعم
   2. لا

أسئلة حول تاريخ الحساسية التنفسية

9. هل يوجد أحد من أفراد عائلتك يعاني من الحساسية التنفسية (تم تشخيصه عن طريق الطبيب) ؟ * (اختر خيار واحد فقط)

   1. نعم
   2. لا

10. هل لديك تاريخ مرضي بالحساسية التنفسية (تم التشخيص عن طريق الطبيب) ؟ * (اختر خيار واحد فقط)

   1. نعم
   2. لا

11. هل تعرف الإرشادات السعودية الخاصة بالسيطرة على الحساسية التنفسية؟ * (اختر خيار واحد فقط)

   1. نعم
   2. لا

أسئلة حول أسباب الحساسية التنفسية

12. ماهو نوع الحساسية التنفسية التي تعاني منها (بناءً على تشخيص الطبيب)؟ * (اختر جميع الاحتمالات المنطبقة)

   1. الربو الشعبي
   2. حساسية الأنف
   3. نوع آخر من الحساسية

13. أي من هذه العوامل هو السبب المحتمل للحساسية التنفسية لديك؟ * (اختر جميع الاحتمالات المنطبقة)

13.1 التلوث الهوائي
13.2 التدخين
13.3 الحساسية الجلدية
13.4 إلتهابات الجهاز التنفسي
13.5 طبيعة العمل
13.6 نوع الطعام
13.7 أسباب وراثية
13.8 أخرى (أذكرها)
13.9 لا أعرف

أسئلة حول أعراض الحساسية التنفسية

14. أي من الأعراض التالية للربو الشعبي تعاني منه؟ * (اختر جميع الاحتمالات المنطبقة)

14.1 صوت مزعج أثناء التنفس
14.2 صعوبة بالتنفس في الطقس البارد
14.3 سعال متكرر
14.4 ضيق في التنفس أثناء التمارين الرياضية أو بالقرب من الحيوانات الأليفة
14.5 صعوبة في النوم أو الاستيقاظ من النوم في الليل بسبب الربو الشعبي
14.6 لا أعاني من الربو الشعبي

15. أي من الأعراض التالية لحساسية الأنف تعاني منه؟ * (اختر جميع الاحتمالات المنطبقة)

15.1 العطس أثناء التمارين الرياضية أو حول الحيوانات الأليفة
15.2 سيلان الأنف
15.3 حكة العيون، احمرار العيون، دموع العيون
15.4 صعوبة بالنوم أو الاستيقاظ من النوم في الليل بسبب حساسية الأنف
15.5 لا أعاني من حساسية الأنف

أسئلة حول علاج الحساسية التنفسية

16. هل تناولت سابقاً أدوية لعلاج الحساسية التنفسية *(اختر خيار واحد فقط)

1. نعم
2. لا

أسئلة حو توفير الأدوية لعلاج نوبات الحساسية التنفسية

17. هل امتثلت بتناول هذه الأدوية لعلاج الحساسية التنفسية؟ * (اختر خيار واحد فقط)

1. نعم - دائماً
2. لا - أبداً
3. نعم - أحياناً

18. أي من الطرق التالية استخدمتها لعلاج حالة الحساسية التنفسية لديك؟ * (اختر جميع الاحتمالات المنطبقة)

18.1 استنشاق موسعات الشعب الهوائية
18.2 موسعات الشعب الهوائية عن طريق الفم
18.3 استنشاق بخاخات الستيرويد
18.4 تناول الستيرويد عن طريق الفم
18.5 تناول الستيرويد عن طريق الوريد
18.6 جهاز الرذاذ لحالات الطوارئ
18.7 التنويم في المستشفى

19. أين تتناول هذه الأدوية لعلاج حالة الحساسية التنفسية؟ * (اختر خيار واحد فقط)

1. في المنزل
2. في المركز الصحي
3. في المستشفى
