# Supplementary material for: Prevalence and associated factors of respiratory allergies in the Kingdom of Saudi Arabia: A cross-sectional investigation, September–December 2020
Source: PLoS One. 2021 Jun 23;16(6):e0253558. doi: 10.1371/journal.pone.0253558 (PMC8221486; doi:10.1371/journal.pone.0253558)
Supplement: S2 Appendix — (PDF) [file pone.0253558.s002.pdf]

Respiratory allergies in the Qassim region, Saudi Arabia

Questions about personal information

1. Where are you living? * *Mark only one.*

    1. Buraydah
    2. Unaizah
    3. Alrass
    4. Other area in Qassim region
    5. Outside Qassim region

2. What is your nationality? **Mark only one.*

    1. Saudi
    2. Non-Saudi

3. What is your gender? * *Mark only one.*

    1. Male
    2. Female

4. What is your age (years)? * *Mark only one.*

    1. Under 15
    2. 15 - 30
    3. 31 - 45
    4. Over 45

5. What is your job? * *Mark only one.*

    1. Student
    2. Employee
    3. Worker
    4. Other job

6. Does your work related to health sector? **Mark only one.*

1. Yes
2. No

7. What is your educational level? * *Mark only one.*

1. Secondary or below
2. Diploma or Bachelor
3. Postgraduate

8. Do you have a present or past history of smoking? * *Mark only one.*

1. Yes
2. No

Questions about history of respiratory allergies

9. Do you have a family member suffering from respiratory allergies (diagnosed by a doctor)? * *Mark only one.*

1. Yes
2. No

10. Do you have a personal history of respiratory allergies (diagnosed by a doctor)? **Mark only one.*

1. Yes
2. No

11. Do you know about KSA guidelines for respiratory allergy control? * *Mark only one.*

1. Yes
2. No

Questions about causes of respiratory allergies

12. Which type of respiratory allergy you suffer from (based on a doctor's diagnosis)? * *Mark all that applies.*

1. Bronchial asthma
2. Allergic rhinitis
3. Other type of allergy

13. Which of the following is a possible cause that developed your case of respiratory allergic diseases? * *Check all that apply.*

13.1 Air pollution
13.2 Smoking
13.3 Atopy
13.4 Respiratory tract infections
13.5 Occupation
13.6 Diet
13.7 Genetic predisposition
13.8 Others (please specify)
13.9 I do not know

Questions about symptoms of respiratory allergies

14. Which of the following symptoms of bronchial asthma you are suffering from? * *Check all that apply.*

14.1 Noisy breathing
14.2 Difficult breathing in cold weather
14.3 Repeated coughing
14.4 Shortness of breath during exercise or around pets
14.5 Awakenings at night/difficulty sleeping due to bronchial asthma
14.6 I do not suffer from bronchial asthma

15. Which of the following symptoms of allergic rhinitis you are suffering from? * *Check all that apply.*

15.1 Sneezing during exercise or around pets
15.2 Runny nose
15.3 Red, watery, or itching eyes

15.4 Awakening at night/difficulty sleeping due to allergic rhinitis

15.5 I do not suffer from allergic rhinitis

Questions about treatment of respiratory allergies

16. Did you take a medication for treatment of respiratory allergy? **Mark only one.*

1. Yes
2. No

Questions about provision of treatment for attacks of respiratory allergies

17. Did you comply with these medications for treatment of respiratory allergy? * *Mark only one.*

1. Yes- always
2. No- never
3. Yes- sometimes

18. Which of the following methods is used to manage your case of respiratory allergy? * *Check all that apply.*

18.1 Inhaled bronchodilator sprays

18.2 Oral bronchodilators

18.3 Inhaled steroid sprays

18.4 Oral steroids

18.5 IV steroids

18.6 Emergency nebulization

18.7 Stayed at the hospital

19. Where can you get this type of medication? * *Mark only one.*

1. At home
2. At the primary health care center
3. At the hospital
